# Microglia and the Aging Brain: Are Geriatric Microglia Linked to Poor Sleep Quality?

**DOI:** 10.3390/ijms22157824

**Published:** 2021-07-22

**Authors:** Mohammed E. Choudhury, Kazuya Miyanishi, Haruna Takeda, Junya Tanaka

**Affiliations:** 1 Department of Molecular and Cellular Physiology, Ehime University Graduate School of Medicine, Shitsukawa, Toon 791-0295, Ehime, Japan; 2 International Institute for Integrative Sleep Medicine (WPI-IIIS), University of Tsukuba, Tsukuba 305-8575, Ibaraki, Japan; miyanishi.kazuya.xp@alumni.tsukuba.ac.jp; 3 Department of Gene Expression Regulation, Institute of Development, Aging and Cancer, Tohoku University, Aoba, Sendai 980-8575, Miyagi, Japan; takeda.haruna.r2@dc.tohoku.ac.jp

**Keywords:** aging, microglia, synapse, sleep, circadian rhythm

## Abstract

Poor sleep quality and disrupted circadian behavior are a normal part of aging and include excessive daytime sleepiness, increased sleep fragmentation, and decreased total sleep time and sleep quality. Although the neuronal decline underlying the cellular mechanism of poor sleep has been extensively investigated, brain function is not fully dependent on neurons. A recent antemortem autographic study and postmortem RNA sequencing and immunohistochemical studies on aged human brain have investigated the relationship between sleep fragmentation and activation of the innate immune cells of the brain, microglia. In the process of aging, there are marked reductions in the number of brain microglial cells, and the depletion of microglial cells disrupts circadian rhythmicity of brain tissue. We also showed, in a previous study, that pharmacological suppression of microglial function induced sleep abnormalities. However, the mechanism underlying the contribution of microglial cells to sleep homeostasis is only beginning to be understood. This review revisits the impact of aging on the microglial population and activation, as well as microglial contribution to sleep maintenance and response to sleep loss. Most importantly, this review will answer questions such as whether there is any link between senescent microglia and age-related poor quality sleep and how this exacerbates neurodegenerative disease.

## 1. Introduction

Due to advanced medical support, there are more individuals aged >65 years than children aged <5 years in the world. A growing body of evidence shows that the incidence of age-related diseases, which are mostly neurodegenerative diseases, is increasing and will increase in the future. Compared with other organs, brain aging draws the most attention in clinical settings because senescent changes are irreversible and severely impact the daily lives of older people and caregivers [1]. Aging is the leading predisposing factor of most common neurodegenerative diseases, such Alzheimer’s disease (AD) and Parkinson’s disease (PD) [2], and sleep disturbances are considered additional risk factors underlying the pathophysiology of these neurodegenerative diseases. Regarding AD, a study showed that older women who slept <5 h per night over a 2 year period had worse cognitive performance than those who slept 7 h per night [3]. Furthermore, the sleeping brain efficiently cleanses β-amyloid (Aβ), a notorious protein for AD brain, highlighting a critical function of the sleep/wake cycle for metabolic detoxification for this disease [4]. Regarding PD, patients with idiopathic rapid eye movement (REM) sleep behavior disorder (IRBD) showed remarkably reduced ^18^F-DOPA uptake in a positron emission tomography (PET) imaging study, indicating an association between this sleep disorder and PD pathogenesis [5]. In addition to sleep disturbances in neurodegenerative diseases, prolonged sleep latency, increased transition frequencies to lighter stages of sleep and wakefulness, more time spent awake after sleep onset, more fragmented sleep, and less time in slow-wave sleep are common sleep patterns of healthy elderly individuals [6]. It has been postulated that, with the growing aging population, the burden of poor-quality sleep will continue to rise. Therefore, a better understanding of the pathophysiology at the molecular and cellular levels is necessary to obtain the mechanistic and therapeutically relevant insights into poor sleep in elderly people and in patients with age-related neurodegenerative diseases.

**Figure 1:**
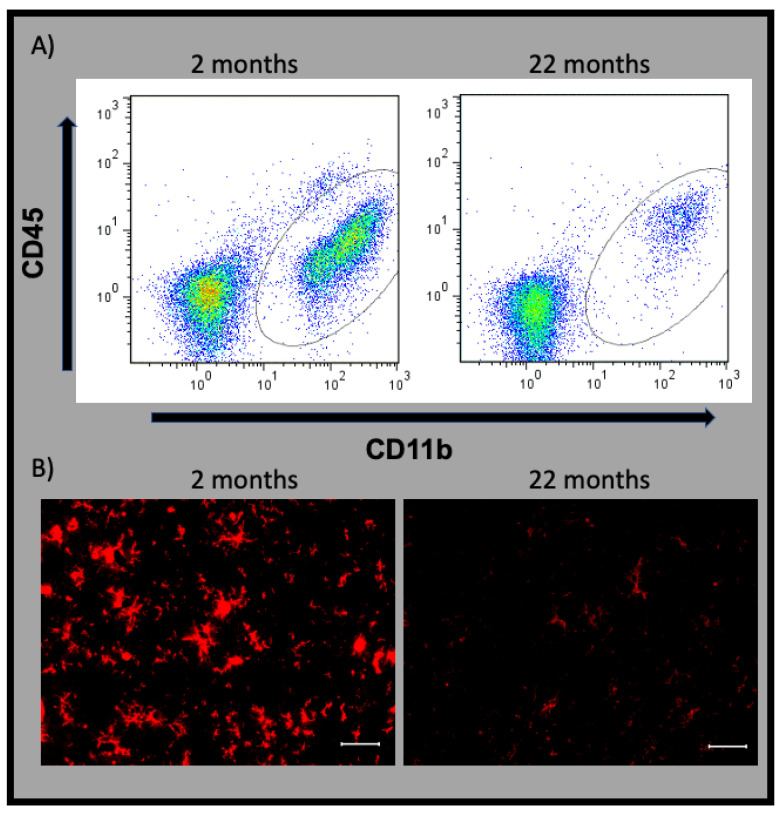
Age-induced drastic decline of the microglial cell population, as revealed by flow cytometry with CD45 and CD11b staining. Microglia are identified (**A**) within gray circles and (**B**) with immunofluorescent Iba1 staining (red). Prefrontal brain tissue of 2- and 22-month-old male Wistar rats was used for flow cytometry and immunofluorescence, as described in our previous study [12]. Brain sampling was performed at Zeitgeber time (ZT) 7. Scale bar = 40 µm.

Unusual synaptic structures, such as decreased synaptic density and terminals, are noted in the neurons of aged brains [7]. A study using the highly validated radiotracer ^11^C-PK11195-PET to target an 18-kDa translocator protein, which was overexpressed in activated microglia [8], showed profound neuroinflammation, in addition to neuronal changes, in patients with IRBD [5]. In brain neuroinflammation, microglia and innate immune cells are key modulators driving neurodegeneration [9,10]. However, increasing evidence has shown the involvement of microglial cells in maintaining normal homeostatic function of the brain through normal visual function maintenance, sleep maintenance, and diurnal body temperature regulation [11,12,13]. In addition, the number of microglial cells is dramatically decreased in aged mice [14], a finding that we also found in aged rat brain (Figure 1). Furthermore, microglia in the aged brain show dystrophic, gnarling, and beading features with an increased secretory profile [15]. This review summarizes the current knowledge of functional and phenotypic properties of senescent microglia and highlights the contribution of senescent microglia to sleep abnormalities among the elderly population.

## 2. Microglia and Their Role in Normal Adult Brain

Pio del Rio-Hortega [16] first introduced microglia as Hortega cells, which were described as invasive, mesodermally oriented, and amoeboid-like brain cells. In the healthy mature brain, microglia are ramified in morphology with a small somata containing fine cellular processes. Due to inherent difficulties in studying this cell, it was conventionally considered to be a resting or immunologically quiescent type of cell, a belief that remained unchanged for a long time until the development of in vivo two-photon laser scanning microscopy in the early 2000s. At that time, there was a growing body of evidence supporting the hypothesis that microglial processes followed neuronal activity patterns. Using two-photon microscopy, Nimmerjahn and his colleagues [17] analyzed microglial cells from a transgenic mouse line in which enhanced green fluorescent protein (EGFP) expression was achieved by inserting its reporter gene in the *CX3CR1* locus, which encodes the chemokine receptor *CX3CR1*. They found that, under physiological conditions, microglia were mostly in a “resting” state, with highly motile processes continuously “patrolling” the surrounding microenvironment. In the process of screening, microglial cells shift their territories and continuously sense the surrounding microenvironment, dynamically interacting with the surrounding elements. Furthermore, increased neuronal activity caused by the exposure to the GABAA receptor antagonist, bicuculline, enhances microglial process sampling. A later study [18] using *Iba1*-EGFP and *Thy1* promoter-GFP mice showed that microglia formed a bulbous contact with synapses and that contact was preferential and specific as microglia extended processes and made bulbous contacts with spines/synapses but did not make any contact with dendritic shafts. Moreover, the suppression of neuronal activity, detected by binocular eye enucleation, tetrodotoxin injection into both retinas, or body temperature reduction decreased microglial contact with synapses at specific regions of the brain. These findings suggest that the microglia–synapse contact is neuronal activity dependent. An extraordinary study by Tremblay and her colleagues [11] on juvenile mice showed that the extracellular space apposing microglia was larger than the space not apposing microglia, suggesting an influence of microglia in the creation of extracellular space. In vivo imaging of *CX3CR1*-GFP/*Thy1*-YFP double-cross transgenic mice demonstrated that dendritic spines close to microglial processes were smaller than the rest of the spine population. Most fascinatingly, two-photon imaging showed the involvement of microglia in controlling the synaptic structures of both axon and dendrites. In response to different brain experiences, microglia were found to change their behavior with the synapse and surrounding neurons, such as the regulation of extracellular spaces, apposition and phagocytosis of synaptic elements, and dynamic interaction with subsets of dendritic spines [11]. Li and her colleagues [19] reported that the surveillance activity of the resting microglia in larval zebrafish was not a seemingly random process but was instructed primarily by local neuronal activity. Microglial processes preferentially navigated toward and simultaneously made bulbous contact with neurons that exhibited higher spontaneous activity. They [19] also demonstrated that a high level of neuronal activity releases a “find-me signal” (ATP as a candidate) for microglia to come close to highly active neurons through sensing the signal. More interestingly, they demonstrated that contact made by microglia relaxes the neuronal activity. Together, these findings indicate that microglia–neuron interactions under physiological conditions are necessary for establishing, maintaining, and guarding neuronal activity necessary for normal daily activities.

## 3. Sleep–Wake Cycle Impact on Microglia–Synapse Interaction

At the onset of sleep, there is a surge in ATP level across several brain regions, including the frontal cortex and hippocampus [20]. As stated in Section 2, ATP is considered an important chemoattractant of microglia, and its breakdown product ADP binds to the *P2Y12* receptor, which is a purinergic receptor expressed by homeostatic microglia [21,22]. The systemic administration of clopidogrel, a *P2Y12* receptor inhibitor, increased the density of cortical dendritic spines during the light phase, suggesting that microglia prune synapses during sleep through purinergic signaling [23]. Krueger and his colleagues [24] reported that the administration of a *P2X* receptor agonist increased non-REM (NREM) sleep and electroencephalographic (EEG) delta power, whereas a *P2X* receptor antagonist suppressed NREM sleep in rat. In the light phase or under anesthesia, microglial processes displayed highly active motility and the phagocytosis of synapses compared with the wake state and dexamethasone, which sedated microglial functions and disrupted sleep [12,25]. The administration of minocycline, a tetracycline-derived antibiotic widely used in reducing microglial phagocytic and inflammatory functions, affected sleep homeostasis in mice [26] and humans [27]. In the sleep–wake cycle, microglia show morphological and molecular alterations, as well as various responses to neuromodulators and cytokines, thus suggesting the possible involvement of microglia in sleep regulation.

## 4. Diurnal Rhythmicity in Microglial Structure

A leading study on this topic by Hayashi and colleagues [23] reported that cortical microglia exhibited diurnal morphological changes in mice that were maintained by clock gene-driven diurnal expression of *P2Y12R*. Morphometrical investigation with skeletonized images reconstructed from Z-stack images of Iba1-immunostained microglia showed pronounced diurnal rhythmicity in cortical microglia that were characterized by more elongated and complex processes during the dark than during the light phase. The administration of the *P2Y12R* inhibitor, clopidogrel, reduced the circadian behavior of microglia process alteration. They also speculated that the release of ATP and glutamate from highly active neurons played a critical role behind the rhythmicity of the microglial process. Recently, we also confirmed such interesting diurnal rhythmicity in microglial morphology, specifically on the size and granularity of cellular somata at the prefrontal cortex, using flow cytometry, in which microglia showed higher forward and side scatter values at the onset of the light phase than those at the onset of the dark phase. These results suggest that microglia at the onset of sleep are larger and more granular than those at the onset of waking [12].

## 5. Diurnal Rhythmicity in Microglial Interactions with Neurons and Neuronal Elements

Over the last decade, microglial interactions with synapses were shown to play crucial roles in the formation, maintenance, and elimination of synapses, and to be involved in neuronal plasticity for learning, memory, and adaptation to enriched or stressful environments. Beyond immunosurveillance, microglia showed diurnal rhythmicity with synapse phagocytosis for maintaining the homeostasis of synaptic strength [12,28,29]. In a time-lapse imaging study on dexmedetomidine (DEX)-sedated mice, microglia showed greater motility and made more frequent contacts with motile cortical dendritic spines [25]. Concomitantly, another study showed the microglia of isoflurane-anesthetized mice had increased process velocity and length, as well as a greater number of intersections and branch points [30]. However, to date, such time-lapse imaging observations still lack the verification of diurnal rhythmicity in microglial motility and interaction with neurons during normal sleep. The sleep–wake cycle has an important role in the determination of synaptic strength, and the strength of both excitatory and inhibitory synapses are reduced during sleep [31]. This finding suggests that wakefulness increases spine density and synaptic strength, whereas sleep reduces them. The pruning of dendritic spines or filopodia in sleep is higher than during wakefulness. It is considered that the downscaling of spine density during sleep reduces the signal-to-noise ratio, bringing favorable inputs in memory consolidation or allowing for new learning during subsequent awake periods. During the sleep process, functional synapses are preserved, whereas nonfunctional ones are eliminated [32,33]. In the brains of adolescent mice, the mean spine density in the cerebral cortex during sleep is lower than that during wakefulness [28]. Likewise, immunoblotting of microglia sorted using flow cytometry from the prefrontal brain tissue showed a higher expression of the synaptic marker synapsin1 in the beginning of the light phase than in the dark phase. This finding indicates that microglia engulf more synapses at the onset of sleep to ready the brain for the next day. Furthermore, these findings support the hypothesis that sleep is critical for microglial control of synapse homeostasis [12].

## 6. Diurnal Rhythmicity in the Expression of Genes Related to Microglial Phagocytosis

The possible involvement of diurnal variation in microglial synapse phagocytosis to maintain synaptic plasticity has been suggested for many years [28]. In our previous work, we demonstrated increased expression of *CD68*, a microglial phagosome-specific marker in the brain, during sleep onset than during wake onset [12]. *CX3CR1*, one of the marker genes expressed in the microglia of mice and humans, is implicated in numerous microglial functions. *CX3CR1* expression was linked to synapse elimination [34,35], and high expression of *CX3CR1* was found at the onset of sleep. Moreover, the phagocytic molecules related to the “eat me” signal, such as complement protein *C3*, *C1qB*, *milk fat globule-EGF factor 8 protein* (*MFG-E8*), *mer tyrosine kinase receptor* (*MerTK*), growth arrest-specific protein 6 (*GAS6*), and *protein S*, were diurnally changed and found at high levels at the onset of sleep. Similarly, levels of the mRNA encoding *matrix metalloproteinase 2 (MMP2)*, which microglia commonly use in remodeling events in the surrounding cellular region, also showed a high pattern of expression change at the onset of sleep [12]. Additionally, diurnal changes of cathepsin S mRNA expression were also observed in mice, with cathepsin S used by microglia for the degradation and modification of extracellular matrix (ECM) molecules [28].

## 7. Diurnal Rhythmicity in the Expression of Genes Related to Microglial Inflammation

Rhythmic regulation occurs at least partly at the transcriptional or posttranscriptional level because transcripts of different cytokines show a 24-h rhythm [36]. The induction of the cytokines *IL-6*, *IL-12*, *CCL2*, and *CCL5* from macrophages was found to be greater in mice challenged with the *Toll-like receptor 4 (TLR4)* ligand lipopolysaccharide (LPS) at the active phase than at the resting phase [37]. Further, an analysis of the macrophage transcriptome at the level of the transcripts involved in the *TLR4* response and associated pathway showed that 8% of the transcripts varied with the circadian rhythm, highlighting the control of the circadian clock [37]. Therefore, this strict control of the circadian clock on macrophage *TLR4*-related pathways indicates its involvement in immune cells for pathogen recognition and immune response [38]. Hippocampal brain tissue of rat showed higher inflammatory priming for LPS challenge at the resting period than at the active period, and the rhythmic expression of proinflammatory cytokines, such as *IL-1β*, *TNF-α*, *IL-6*, and *IL1R1*, in hippocampal tissues of sorted microglia was seen in hippocampal tissues. Moreover, microglia isolated at the light phase show increased inflammatory priming following LPS stimulation compared to the dark phase [39]. This group also showed that the microglial-mediated inflammatory response in tail shock-exposed rats was higher when the shock was introduced at the resting phase versus the active phase [40].

## 8. Extrinsic Cellular Factors behind Microglial Rhythmicity

A recent study from our laboratory reported higher levels of noradrenaline in prefrontal cortex lysate at the onset of wakefulness than those at the onset of sleep [12]. This change alternatively associated with diurnal rhythmicity of microglial structure and rhythmic expressional changes of a microglial phagocytic marker. In addition, pharmacological manipulation of noradrenaline using reserpine and L-threo-dihydroxyphenylserine changed microglial diurnal rhythmicity [12]. In the context of microglial phagocytosis, we reported that the exposure of glutamate-stimulated rat microglial cells to noradrenaline decreased the expression of *MMP2*, *cathepsin S*, *MFG-E8*, *C1qb*, *MerTK*, *CX3CR1*, and *IRF1* [12], and similar findings were also obtained in LPS-stimulated cells [41]. The diurnal rhythmicity of microglial cells follows the rhythmic changes of noradrenaline concentrations of the prefrontal cortex. More surprisingly, modulating diurnal rhythmicity for noradrenaline contents through shifting light on and off timing induced expressional changes of microglial phagocytic markers for synapse [12]. Stowell et al. [25] examined the effects of DEX on microglia. DEX exerts a sedative action by lowering the release of noradrenaline from the locus coeruleus. They found that DEX-sedated mice showed a robust increase in the size of microglial arborization and enhanced microglial surveillance. Use of the β2-adrenergic receptor selective agonist clenbuterol caused a marked retraction in microglial processes and motility, resembling those seen in awake mice. Moreover, the administration of the β2-adrenergic receptor selective antagonist ICI-118,551 increased ramification and enhanced surveillance of the parenchyma, as seen during DEX-induced sedation. Another study demonstrated the regulation of microglial process surveillance in vivo, where microglia displayed selective specificity toward noradrenergic tonic signaling [30]. The abovementioned study also reported that β2-adrenergic receptor antagonism and toxic insult to locus coeruleus noradrenergic neurons increased microglial process surveillance in awake mice. The effects of noradrenaline signaling in this study, therefore, mirror the findings of the study by Stowell and colleagues [30].

Glucocorticoids are other factors that induce microglial circadian changes in synapse phagocytosis as endogenous glucocorticoid secretion displays a marked effect on cellular circadian rhythm [39,42]. The peak secretion of glucocorticoids is observed early in the morning in humans [43] and in the early evening in mice [44]. Administration of the glucocorticoid dexamethasone suppressed the expression of microglial phagocytic markers at the onset of sleep when these genes’ expressions were found to be high, normally at this period of the day [12]. Overall, these findings indicate that the diurnal rhythmicity of microglia is regulated either in combination or independently by noradrenaline and corticosterone. However, microglial rhythmic behavioral studies using adrenalectomized and locus coeruleus-lesioned animals are needed to verify this hypothesis.

## 9. Intrinsic Molecular Clocks behind Microglial Rhythmicity

The core components of the cellular circadian clock system are expressed in most cells of the body. The components of this machinery are *brain and muscle ARNT-like-1 (BMAL1)*, *circadian locomotor output cycles kaput (CLOCK)*, and *neuronal PAS domain-containing protein-2 (NPAS2)*. Normally, *BMAL1* and *CLOCK* form a dimer that translocates to the nucleus and binds to the enhancer box motifs throughout the genome, thereby controlling the transcription of clock-controlled genes [45,46,47,48]. Genes controlled by the *BMAL1*/*CLOCK* heterodimer include the period circadian regulators (*PER1*–*PER3*) and cryptochrome circadian regulators (*CRY1* and *CRY2*) [46,49,50]. The products of these genes use a feedback mechanism to inhibit their own expression via *BMAL1/CLOCK*. This feedback process takes about 24 h and is very tightly regulated [51,52]. An additional level of regulation, working along with the core clock to establish and regulate the 24 h rhythms, occurs via *REV-ERB/RORα* and *NFIL3* [53]. The components, loops, and circuits of these complex cycles contribute to establish the intrinsic 24 h period of the circadian clock. Like other cells, immune cells, namely, macrophages, dendritic cells, and B cells have functional molecular clocks, which exhibit daily oscillations in the mRNA abundance of canonical clock genes [54]. To the best of our knowledge, Nakazato and colleagues [55] first reported the existence of molecular clock genes in the primary culture of mouse microglia. They also reported the expression of all clock genes, except *CLOCK*, in the cells from the BV2 murine microglial cell line. In the healthy brain, there is the expression of intrinsic molecular clock genes, such as *PER1*, *PER2*, *REV*-*ERBα*, and *BMAL1*, in the microglia of cortical and hippocampal tissues [28,39]. In mice, except for BMAL1, most of the mRNAs encoding the molecular clock are found at high levels at night, specifically Zeitgeber time (ZT) 14 than at ZT2 [28]. In rats (another nocturnal mammal), the expression of *REV-ERBα* and *BMAL1* showed a similar pattern as that in mice; however, mRNA expression of *PER1* and *PER2* shows an opposite pattern [39]. The explanation behind these apparent discrepancies between results is unclear but may include potential differences in brain region- and animal-specific expression patterns of the molecular clock in microglial cells.

## 10. Alteration of Clock Genes Changes the Microglial Phenotype

Circadian rhythms are involved in the regulation and maintenance of the immune response [46]. The mechanism behind the circadian control of microglial inflammatory cytokine secretion remains unclear in most cases. Emerging evidence supports the understanding that the core components of circadian clock machinery, mainly *BMAL1*, *CLOCK*, *REV-ERBα*, and *RORα*, are engaged in the regulation of inflammatory functions. Basal oscillations of the expression of inflammatory genes are regulated by *BMAL1* [56]. *IL-6* is an important proinflammatory cytokine of microglial cells and, in response to LPS, *IL-6* is produced in primary microglial cell cultures and BV2 microglial cells. However, the release of *IL-6* by microglia and BV2 cells deficient in *BMAL1* was significantly lower than that of normal cells [57]. Moreover, *BMAL1*-knockout mice showed decreased expression of *IL-1β* and *Nox2* [58]. The rhythmic modulation of inducible gene expression relies on interference with transcription factor *NF-κB*, which is a major transcriptional activator of inflammation [59]. The core circadian protein *CLOCK* favors *NF-κB*-mediated transcription, and the sequestration of *CLOCK* by *BMAL1*-induced rhythmic repression may be via the expression of *NF-κB*-mediated inflammatory genes such as *IL-6* [59]. In the mouse brain, genetic deletion of *REV-ERBα* increased microglial expression of *Iba1*—a marker of microglial activation. *REV-ERBα*-deleted microglial cells showed decreased microglial branching and increased *CD68*, *IL-6*, *CCl2*, and *TNF*-α expressions [60]. Moreover, *REV-ERBα*-deficient mice showed increased microglial phagocytosis of synapses in the CA3 region of the hippocampus [29]. Similarly, genetic or pharmacological suppression of *REV-ERBα* in microglial cells showed decreased uptake and clearance of Aβ [61]. Administration of the *REV-ERBα* agonist, SR9011, decreased the inflammatory response of primary microglia [62]. *REV-ERBs* contribute to a negative feedback loop of the cellular clock repressing a subset of inflammatory genes in a signal-dependent manner by inhibiting enhancer-specific transcription [63]. Therefore, microglia with dysregulated internal clocks are capable of perpetuating neurodegeneration in a feedback loop of inflammatory signaling.

## 11. Senescent Microglia

Aging is a physiological process characterized by a reduction in brain performance, reduction in synaptic plasticity, and alterations in neurotransmission, as well as receptor availability in the central nervous system (CNS) that may affect cognitive performance [64,65]. In human samples, microglia in the aged brain show dystrophic morphology characterized by a slight enlargement of size, the distinct loss of fine branches, and the formation of cytoplasmic spheroids, gnarling, beading, and fragmentation [15]. On the basis of the results from immunohistochemical and morphological analyses, microglia of aged mice showed structural alteration characterized by decreased arbor area and increased arbor circularity index [66]. Using flow cytometry, we recently described such morphological changes in rats, where microglia from aged rat brain were remarkably larger in size and more granular. In addition to morphological changes, aged microglia show increased expression of the phagocytosis-related molecules, *CD11b*, *CD68*, and *NG2*, as well as the proinflammatory phenotype-related markers, *CD86* and *CD45* [67]. Moreover, retinal microglia in aged mice exhibit decreased motility and reactivity in surveying the surrounding CNS microenvironment and the ability to respond to injury and inflammatory conditions [68]. In addition to the phenotypic changes, Zöller and colleagues showed, using immunohistochemistry, that the number of *Iba1*-positive cortical microglia was drastically decreased in aged mice [14]. Our flowcytometric and immunofluorescence microscopic observations of aged rat brain (Figure 1) showed similar findings, with a reduced population of microglia over the total number of live cells in the rat prefrontal cortex. Furthermore, profound modifications in the transcriptome profile, secretome, morphology, and phagocytic activity of aged microglia are associated with the housekeeping and defensive functions of microglia [69]. In addition, the functional properties of senescent microglial changes are sex specific [70], and changes in energy metabolism are considered responsible for their reduced phagocytotic capacity [71].

## 12. Diurnal Rhythmicity of Senescent Microglia

Weak biological rhythmicity in aged brain can occur for different reasons. First, the deterioration of sense organs, mainly retina, results in a decreased input to the suprachiasmatic nucleus [72]. Second, weakened suprachiasmatic nucleus outputs are associated with aging [73,74,75]. In the milieu of the external clock, there is also significantly smaller mesor and amplitude of the 24 h rhythm of noradrenaline content in an aging rat [76]. Furthermore, decreased noradrenaline caused by senescent degeneration of the locus coeruleus is implicated in the pathologic activation of microglia in AD [77]. In addition to noradrenaline, in the rat, glucocorticoids affect microglial morphology and reduce microglial activation [78] and phagocytosis [79]. Moreover, continuous low levels with no time-of-day variations in adrenal corticosterone were observed in aged rat [80]. As mentioned above, the rhythmicity of microglial cells depends on the patterns of noradrenaline, as well as corticosterone, and these patterns are compromised in the aged brain.

**Figure 2:**
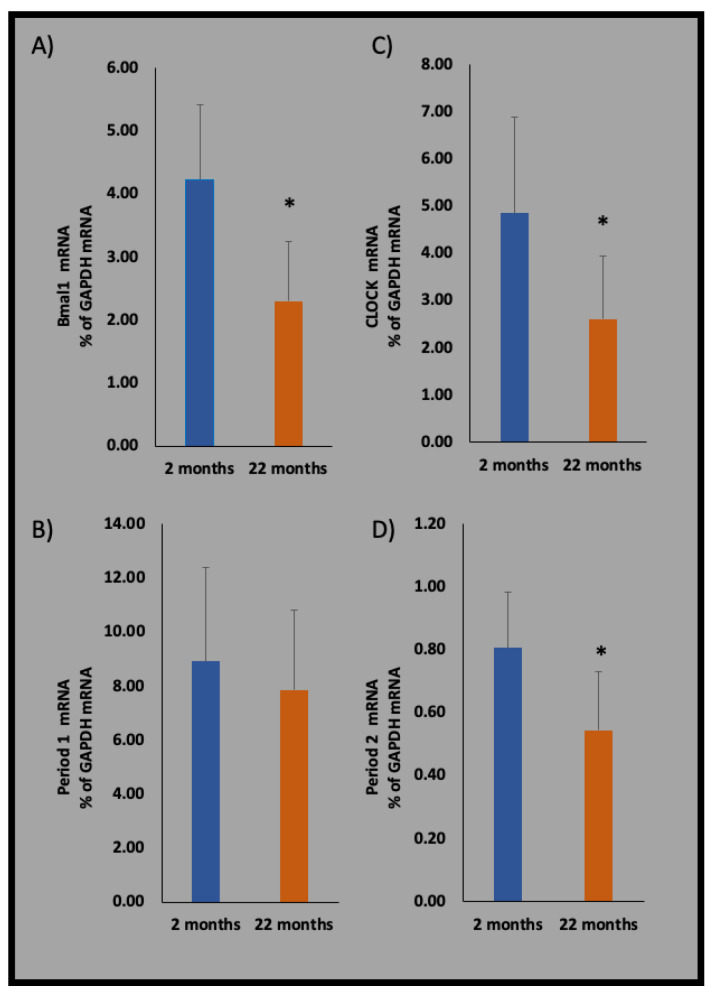
mRNA expression changes of: (**A**) *BMAL1*, (**B**) *PER1*, (**C**) *CLOCK*, and (**D**) *PER2* in flow-sorted microglia in the prefrontal cortices of 2- and 22-month-old male Wistar rats (both *n* = 6). Samples were collected at ZT7. Microglia were sorted from the prefrontal cortex as we described previously [26]. RNA extraction and reverse transcription for quantitative polymerase chain reaction (qPCR) were conducted using a SuperPrep™ II Cell Lysis & RT Kit (Toyobo, Osaka, Japan). qPCR was performed as described previously [26]. The expression of clock genes was suppressed in senescent microglial cells. Data are expressed as mean ± standard deviation (SD). * *p* < 0.05, with unpaired two-tailed *t*-test.

Regarding the intrinsic molecular clock, the aged rat brain displays diminished rhythms in the regulation of inflammatory cytokines. More specifically, aged hippocampal microglia displayed suppressed rhythmic expression of *PER1* and *PER2*; however, they displayed rhythmicity with *BMAL1* and *REV*-*ERB* α expression [42]. Moreover, decreased mRNA expression levels of *BMAL1*, *CLOCK*, and *PER2* were found in the aged microglia of the rat prefrontal tissue (Figure 2). The decreased expression of *BMAL1* may be associated with the increased proinflammatory nature of aged microglia [42].

## 13. Sleep Characteristics in Aged Rodents

In humans, impairments in REM sleep occur more frequently with aging, especially at the age of ≥80 years [81,82]. Aged mice share several common sleep pattern features with elderly humans, but with a single major difference: there is an increase in the delta power in the frontal cortex of aged mice, whereas it is decreased in elderly humans [83]. Several studies have shown that the amplitude, timing of circadian rhythms, sleep quality, and waking performance are affected in aged mice [73,84,85,86,87]. Moreover, aged mice show increased sleep fragmentation and propensity in the active phase with less prominent diurnal rhythm in the sleep–wake cycle [73,87]. In addition, aged mice exhibit increased NREM sleep and decreased waking, especially in the dark period [83,88].

**Figure 3:**
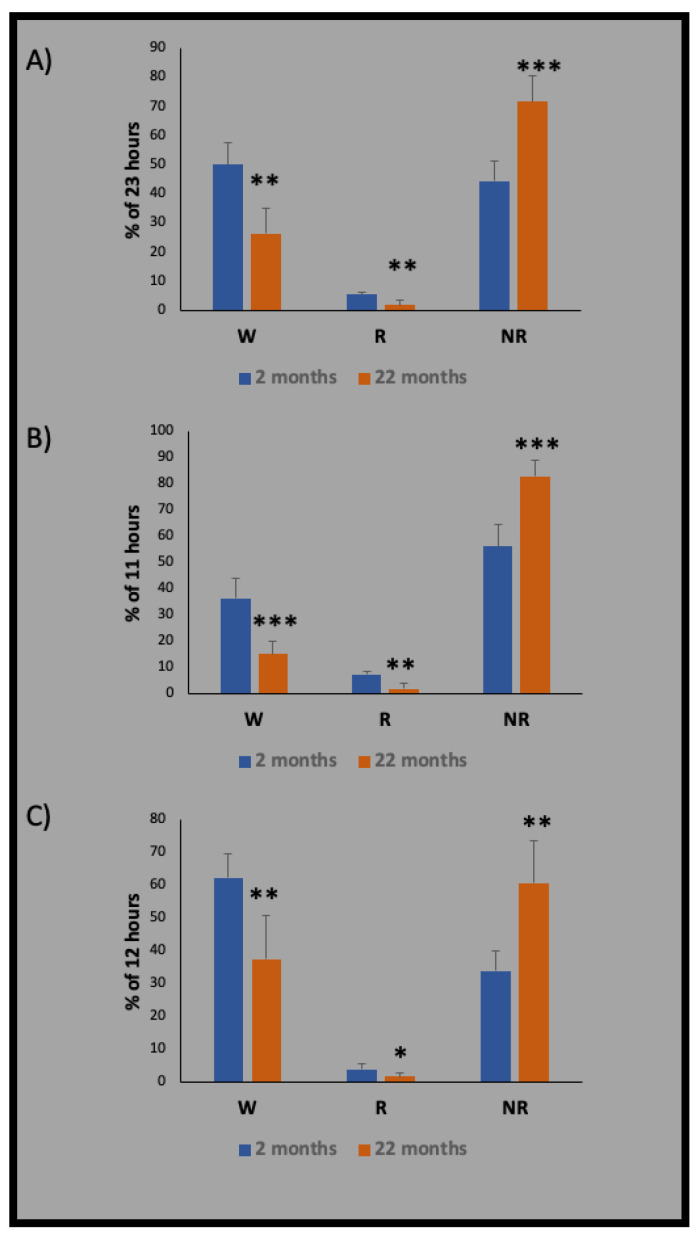
Effects of age on sleep, as investigated by electroencephalography/electromyography recordings on 2- and 22-month-old male Wistar rats (blue and orange column, respectively). In aged rats, there was a markedly increased sleep period and reduced wakefulness in both light (resting phase) and dark (active phase) periods. Recordings were performed over: (**A**) full-day period (ZT1–ZT23), (**B**) light phase (ZT1–ZT11), and (**C**) dark phase (ZT12–23). Data are expressed as mean ± SD (*n* = 5). * *p* < 0.05, ** *p* < 0.01, and *** *p* < 0.0001, with unpaired two-tailed *t*-test. The recording procedure was described in our previous study [26].

In aged rats, sleep quality was moderately compromised with a decrease in the percentage of total sleep time spent in paradoxical (REM) sleep, a decrease in the length of sleep bouts, an increase in the number of sleep bouts, and a decrease in the amplitude of the diurnal rhythm of sleep [89]. However, only reduced sleep time and shortened sleep bouts were found in aged rats later in this study [90]. A previous study showed reductions in high-voltage NREM sleep, the mean length of sleep bouts, and the duration of REM onset in aged rats [91]. Similarly, we also found excessive sleep at the active phase of aged rats and a significant reduction in REM sleep (Figure 3).

## 14. Senescent Microglia and Their Impact on Sleep

On the basis of the findings from an antemortem study on sleep fragmentation and a postmortem immunohistochemical study on neocortical microglial morphology, a strong association was found between the activation of senescent microglia and sleep fragmentation in elderly people, as a greater proportion of morphologically activated microglia was associated with greater sleep fragmentation [92]. First, in the aged brain, there is a massive decline of microglial cells (Figure 1), and a study using a *CX3CR1*-DTR transgenic Wistar rat model for microglial cell depletion showed pronounced disruption in diurnal temperature, metabolism, and activity measures [13]. Second, the expression of *TNF*-α and *IL-1β* in sorted microglia from hippocampal tissues was diurnally regulated in young rats but not in aged rats, and the expression of these cytokines was high in aged rats [42]. In rats, the expression of *TNF-α* and *IL-1β* followed a circadian oscillation in the brain, with higher concentrations associating with greater sleep need [93,94,95,96]. Moreover, *TNF-α* and *IL-1β* expression was high, concomitant with increased NREM sleep, in the condition of sleep loss in rats and rabbits [97,98,99]. The administration of *TNF-α* and *IL-1β* increased sleep need, which was characterized by an increase in EEG delta power during NREM sleep, and also increased NREM sleep duration [33,100,101,102,103,104]. Furthermore, the increased release of inflammatory cytokines, such as *IL-1β*, *IL-6*, and *TNF-α*, was shown to induce sleep in mice [105,106]. These findings highlight the key role of the high levels of cytokines in aged microglia toward greater sleep needs in the aged population. Finally, as noted before, the reduced expression of molecular clock genes, mainly *BMAL1*, in aged microglia may also be associated with the microglial proinflammatory release of cytokines and induce excessive sleep [107].

## 15. Effect of Sleep Loss on Microglial Senescence

Due to the work patterns and workload of modern society, people are accustomed to prolonged wakefulness and sleep insufficiency; however, this habituation is now recognized as a serious public health issue [108,109]. Increasing evidence from animal and human studies indicates that sleep is essential for proper cognitive performance in various domains, such as focus, executive function, learning, and memory development, by restoring energy balance and optimizing synaptic plasticity and homeostasis [110,111,112,113,114,115]. In the elderly, there is an increased incidence of poor sleep, where the total sleep time decreases and the number of awakenings after sleep onset increases [6]. Furthermore, previous studies have associated degenerative neurological diseases with exaggerated neuroinflammatory response [84,116,117,118]. In fact, a recent review showed that sleep loss induced profound microglial activation and increased a cytokine/chemokine surge in cortical and hippocampal tissues in adolescent, adult, and aged mice/rats [119]. Regarding microglial genes associated with phagocytosis, increased expression of *CD11b*, *NG2*, and *CD68* was common in the microglia of aged brain [14,67]; similar findings were obtained in the hippocampal microglia of adult rats after 5 days [120] and 12 h [121,122] of sleep deprivation. Sleep loss may induce or accelerate microglial senescence.

## 16. Effects of Commonly Used Drugs for Sleep Disorders on Microglial Performances

Classically, sedatives are thought to affect neural functions; however, care should be taken, as they can also regulate microglial activations and phenotypic alterations [123]. Among the clinically approved drugs, benzodiazepines are the most extensively investigated and are widely used in the treatment of sleep disorders [124]. They exert hypnotic/sedative effects through the GABA receptor and also bind to translocator protein (18 kDa; TSPO). TSPO, or peripheral benzodiazepine receptor, has been studied as a biomarker of reactive gliosis [125]. TSPO expression is low in normal physiological conditions, but it is significantly upregulated in inflammatory states, including age-related neurodegenerative diseases (e.g., AD and PD) [126]. A study with ^11^C-PK11195-PET showed increased TSPO expression in microglia [127]. TSPO ligands decreased microglial neuroinflammation in vitro and in vivo, demonstrating that TSPO negatively regulates inflammation in microglia [127,128]. Anti-inflammatory effect of TSPO is at least partly mediated by *NF-kB* deactivation [128]. Z-drug (i.e., zolpidem, zopiclone, and zaleplon) is another type of GABArgic drug. These drugs activate GABA signals without binding to benzodiazepine-binding sites of GABAA receptor. Although effects of z-drugs on microglial cell lines have not been investigated thoroughly, they might also act as TSPO ligands [129]. However, those GABAergic reagents, especially benzodiazepines, are not recommended for elderly people because of the greater risk of adverse effects such as fractures, cognitive decline, and dependence. Thus, the clinical use of non-GABAergic reagents is increasing [130]. Among them, melatonin receptor agonist ramelteon and orexin receptor antagonist suvorexant show safety profiles in the elderly [129]. Ramelteon inhibited the activation of astrocytes and microglia in a mouse model of traumatic brain injury. Furthermore, it exerts anti-inflammatory and antioxidative properties by accumulation of nuclear factor erythroid 2-related factor 2 [131]. To our knowledge, no studies have been conducted describing the relevance of suvorexant and microglial activity.

Collectively, some hypnotics/sedatives show anti-inflammatory properties in microglia and have therapeutic implications in neuroinflammatory disorders. Although these neuroinflammatory diseases have higher incidence in the elderly, how the drugs alter microglial activity and physiological functions when administrated to healthy young/aged brains needs further investigation. Other than acting as a sedative, L-serine as a precursor of other amino acids, such as glycine, was found to potentiate microglial functions in vitro [132]; a clinical study has shown that the consecutive ingestion of L-serine is effective in the treatment of patients with poor sleep [133]. Considering the involvement of microglia in the maintenance of sleep, identifying more agents such as L-serine for nourishing and reprogramming microglial cells in aged brain demands further attention for treating age-related poor sleep quality.

## 17. Conclusions

**Figure 4:**
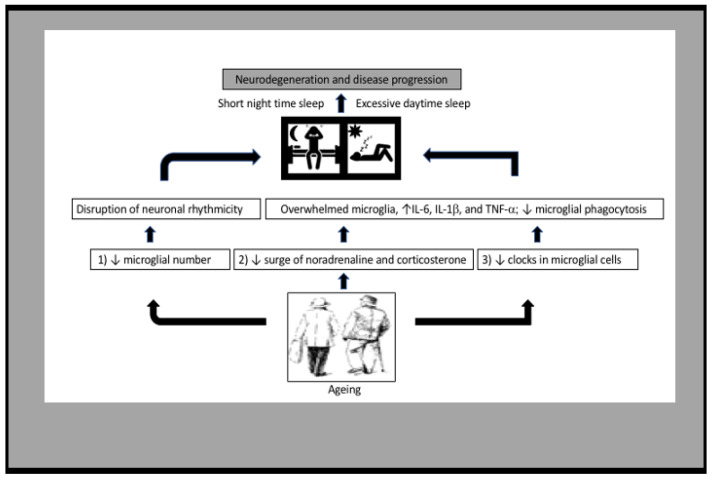
Schematic of microglial contribution in poor-quality sleep in the elderly: (1) the marked decline in microglia disrupts the circadian system of neuronal activity. (2) Decreased concentrations of noradrenaline and corticosterone affect microglial homeostasis. (3) The abnormal rhythmic pattern and/or decreased expression of clock genes drive microglia toward the inflammatory phenotype. The consequences of these three events in microglial cells ultimately affect sleep patterns by altering cellular rhythmic function and increasing proinflammatory cytokine release in aging.

The mechanism narrating age-related impairments in sleep is now being revealed. On the basis of a recent study on microglial phagocytosis of synapses during sleep in healthy adult brain [12], we conducted this review study to answer whether senescent microglia should be considered in understanding the cause of sleep abnormalities in the elderly population. Our conclusion is that the evidence for such a role is overwhelming—microglia play an important role in sleep regulation and the aged brain suffers a major loss of the microglial population and its associated function. In this review, we provided robust evidence on the intrinsic and extrinsic cellular factors affecting microglial rhythmicity, such as corticosterone and noradrenaline (Figure 4). However, future studies are needed to clarify the factors that play a critical role in microglial dysfunction in aging. Moreover, further studies are warranted in the early stages of dysfunction, so that the manipulation of microglia may improve the compromised circadian rhythms found in the aged brain and ameliorate sleep abnormalities.

## Data Availability

Not applicable.
